# 

**Published:** 1910-12-10

**Authors:** 


**TWO USEFUL HANDBOOKS FOR SECRETARIES.**

HOSPITAL EXPENDITURE: THE COMMISSARIAT. For Hospital Matrons and Managers. 2/6, post free.

"This should prove a valuable handbook to those upon whom rests the responsibility of hospital finance, and should be of interest to the public as giving a fair idea of what this department should and does cost."—*Manchester Courier*.

A LEGAL HANDBOOK FOR THE USE OF HOSPITAL AUTHORITIES. By Leonard Syer Bristowe, M.A. Oxon., Barrister-at-Law. 2/6, post free.

**THE SCIENTIFIC PRESS, Ltd.**
28/29 SOUTHAMPTON STREET, STRAND, LONDON, W.C.

### Fibroids and Pregnancy.

The dangers of fibroid tumours complicating pregnancy have been considerably exaggerated; in the majority of cases labour takes place spontaneously without any complication. Whenever possible allow the pregnancy to proceed to full term; you must consider the life of the child as well as that of the mother, and in cases where undue difficulty is met with in delivery, Cæsarean section is likely to give the best results.—*Dr. G. F. Blacker.*

### Obituary.

Mr. Joseph Henry Bond, L.R.C.P.I., late of Thames, Auckland, New Zealand, aged fifty-four, has died in London. He took his L.R.C.S. in Ireland in 1879 and his L.R.C.P. and L.M. in 1882.

The death is reported at The Circus, Bath, of Mr. Tregenna Biddulph Goss, aged seventy-three. He took his M.R.C.S. in 1863, his L.S.A. in 1864, and studied at the Middlesex and Westminster Hospitals. Formerly he was President of the Bath and Bristol branch of the British Medical Association; he was a member also of the Obstetrical Section of the Royal Society of Medicine. He had been Medical Officer to the Bath Post Office, and also the Partis College, Bath.

The death is reported at Ascot of Mr. Walter Brock de Jersey, M.B. Camb., of Netherton, Guildford, at the age of forty-seven. Mr. Brock de Jersey went up to Cambridge, where he graduated M.B., B.C. in 1890, having taken his B.A. in 1884. He took his diploma in 1889, and studied at St. Thomas's. His appointments included those of senior resident medical officer, house surgeon, and assistant physician at the Evelina Hospital for Sick Children. He was also honorary medical officer at the Royal Surrey County Hospital.

Dr. T. N. Brushfield, F.S.A., formerly Superintendent at Chester and Brookwood Asylums, has died at his home at Budleigh Salterton, in his eighty-first year. He took his M.R.C.S. in 1850, and went to St. Andrews University, where he graduated M.D. in 1862. He studied at the London Hospital, where he became house surgeon. He was a member of the British Archæological and the Medico-Psychological Associations, and was the local Secretary of the Society of Antiquaries. He was also ex-President of the Devonshire Association.

### THE ROYAL HOSPITAL FOR INCURABLES.

THE December number of *Travel and Exploration* contains readable and informative articles describing travel experiences in Brittany, Portugal, and Italy. The exploration article is particularly opportune at the present juncture, as Mr. Herbert Sykes incidentally describes the route which the proposed trunk railway to India is intended to take. The article on Brittany will be of special interest to medical men who are fond, like Hodgkin, of "gadding about the globe." Mr. Frederic C. Stanley has hit upon a new topic in his vivid and pleasantly written description of a banana plantation in South America. In "Armchair Travel" an unusually large number of important travel books are reviewed at considerable length.

Last week Dr. T. M. Ross, a practitioner at Chelsea, sued a firm of solicitors for half a guinea in respect of a medical fee due to him. Dr. Ross attended a woman who had been run over by a van, and subsequently she approached the defendants with a view to recovering compensation. The plaintiff was asked for a certificate regarding her injuries, and this he supplied, but received no payment for it. For the defendants it was contended that Dr. Ross should have sued the woman, and that the defendants were in no sense liable. Judge Selfe, in giving judgment for the defendants (without costs), said it was clear to his mind that they had merely acted as agents for the woman.

At a recent Council meeting of the Royal Agricultural Society it was resolved, on the motion of the Earl of Northbrook, Chairman of the Veterinary Committee, seconded by Mr. G. G. Rea, "That, for the protection of our herds and flocks, and in order to prevent the spreading of foot-and-mouth disease and anthrax, it is of the greatest importance that all ships, waggons, or other vehicles which have carried foreign skins, wool, and other substances likely to bring or spread disease, should be thoroughly disinfected before being used for the purpose of carrying cake, feeding-stuffs, or materials used in the manufacture of feeding-stuffs. Further, that it is desirable, if possible, to prevent the carriage of skins, wool, etc., on the same ships as cake and other feeding materials; or, if they must be carried on the same boats, that adequate precautions should be taken to prevent the contamination of feeding-stuffs." Copies of this resolution were ordered to be forwarded to the Board of Agriculture and the Board of Trade.

### Appointments and Resignations.

Mr. H. J. F. Simson, F.R.C.S., has been elected assistant physician for diseases of women at the West London Hospital.

Mr. Brian O'Brien, for a long period honorary attending assistant surgeon to the Belfast Hospital for Sick Children, has resigned his post.

Mr. Philip A. Reckless, M.R.C.S., L.R.C.P., has been appointed resident medical officer of the Western Dispensary, Rochester Row, Westminster. He has been house surgeon to the Leicester Infirmary, and assistant house surgeon to the Sheffield Royal Infirmary, and was a student at St. Bartholomew's Hospital.

Prince and Princess Liechtenstein, Countess Torby, Dora Countess of Chesterfield, Lady Llangattock, Count and Countess Sizzo-Noris, and the Baroness Christine de Linden have given their patronage to the Hungarian concert in aid of the League of Mercy, which will be held at the galleries of the Royal Society of British Artists, Suffolk Street, Pall Mall, S.W., on December 12.

### Editor's Letter-Box.

### ESSEX COUNTY HOSPITAL.

Dr. Temple Chevallier Paley, M.D., M.R.C.S., of Hillthopp Cottage, Great Malvern, who died on August 28, aged 85, leaving estate valued at £12,868 gross, has bequeathed £250 each to the Additional Curates Society, the Royal Medical Benevolent College at Epsom, the Society for the Propagation of the Gospel, and the Society for the Promotion of Christian Knowledge.

### JOTTINGS.

Owing to want of funds twenty beds are unoccupied or "closed" at the Moorfields Eye Hospital.

The King has sent twenty pheasants for the use of the patients at the London Throat Hospital, Great Portland Street, W.

The King has sent a gift of pheasants to the Royal National Orthopædic Hospital, and to the Hospital for Epilepsy and Paralysis, Maida Vale.

Her Royal Highness the Princess Christian of Schleswig-Holstein has given her patronage to Mrs. Kitto's Free Convalescent Home, Reigate.

A new operation-theatre has been opened at S. Joseph's Hospital, Preston, which is a house of the Sisters of Charity. Their congregation was founded in 1832.

The Huntingdon county memorial to King Edward will take the form of support and improvement for the County Hospital at Huntingdon, which is in need of additions.

The Queen has sent a gift of interesting books for the use of the 230 in-patients of the Royal Hospital for Incurables, Putney Heath, of which institution her Majesty is patron.

Ronuk, Limited, well known to hospital workers who use the floor polish manufactured by the firm, has been granted a royal warrant of appointment as sanitary polish manufacturers to H.M. the King.

At the annual meeting of the Axminster Cottage Hospital a proposal to build a small bungalow cottage-hospital, put forward by Dr. W. Langran, was discussed, and it was agreed that ways and means should be considered.

Sir Squire Bancroft will give his reading of Dickens's "Christmas Carol" on December 14, at Folkestone, in aid of the Royal Victoria Hospital. Other intended readings in December have been abandoned owing to the General Election.

Several prominent actor-managers have kindly undertaken to assist Prince Alexander of Teck by giving special *matinées* in aid of the Prince Francis Memorial Fund of the Middlesex Hospital, and interesting announcements in this connection will shortly be made.

Messrs. Henry Young and Sons, of 12 South Castle Street, Liverpool, publish a very interesting photo-lithograph of the late Miss Nightingale. It is an excellent likeness of the "Lady with the Lamp," and is published at the low price of 6d. net, or suitably framed 1s. 6d. It is likely to be very popular as a Christmas card.

The scheme for a new Isolation Hospital for Hinckley, Leicestershire, put forward by Dr. Robinson, the county medical officer, has been recommended for adoption *in toto* by the Hinckley Urban and Rural District Councils. The total cost in round figures has been estimated at £4,000.

Mr. Lyon H. Maxwell, the new Treasurer of the Royal Southern Hospital, Liverpool, has collected a sum of money sufficient to furnish the new out-patient department, which was opened free of debt on November 21 by Mrs. William Adamson (as stated in The Hospital of November 26, p. 273). By the kindly sympathy and encouragement extended to her husband during the many years he has devoted to the hospital's work, Mrs. Adamson has well earned the appreciation and thanks of the Committee. The ceremony was presided over by the Lord Mayor (Mr. S. Mason Hutchinson). Mr. W. H. Lever, Chairman of the Liverpool School of Tropical Medicine, took part in the proceedings, and Mrs. Lever afterwards declared open a new electric lift generously presented by her husband.

Wolverhampton's Hospital Saturday Special Committee has decided to perpetuate King Edward's memory locally by erecting a new wing in connection with the hospital. The Committee asks working-men to contribute a penny a week for three months, and is issuing special "penny brick books."

Thanks to the scheme prepared by Dr. Goodwyn, Bovey Tracey, Devon, is to have a cottage hospital. A house is to be rented and the district nurse installed as matron, with an assistant or wardmaid. The scheme is expected to cost £20 a year; this sum virtually having been guaranteed, the Nursing Association will be responsible for the hospital.

The Lord Mayor's Bristol Hospital Sunday Fund has collected during the past twelve months £2,031. The fund has been in existence for thirteen years, but only twice before has the annual total exceeded £2,000—namely, in 1905 and 1906. Mr. E. G. Swann has been re-elected hon. treasurer, and Mr. Mortimer Harley hon. secretary for the year.

The fifty-fifth annual festival dinner of the Poplar Hospital for accidents, Poplar, E., will be held at the Holborn Restaurant on Wednesday, December 14, at 7 p.m. precisely. Sir Owen Philipps, K.C.M.G., M.P., Chairman of the Royal Mail Steam Packet Company and Vice-Chairman of the Port of London Authority, will be in the chair.

Lady Hardinge of Penshurst has sent from Government House, Calcutta, a large box of dolls and toys for the Christmas festivities in the Children's Ward of the Metropolitan Hospital. The box was accompanied by a letter, in which Lady Hardinge says: "Please tell the children that I think of them and wish all the patients a very happy Christmas."

Prince Alexander of Teck, Chairman of the Weekly Board of the Middlesex Hospital, has received a letter from Mr. J. W. Gifford, of Chard, Somerset, announcing a present of 40 milligrams of radium to the Cancer Research Laboratories of that institution. At current rates this quantity of radium, weighing approximately one seven-hundredth of an ounce, is worth about £600.

The hundred-and-first anniversary of the Derbyshire Royal Infirmary was celebrated by the attendance in state of the Mayor (Sir Thomas Roe, M.P.) and of the Town Council at All Saints' Church. His late Majesty King Edward was one of the life governors of the infirmary, which he visited in 1872. Miss Florence Nightingale also was a subscriber for over thirty years to its funds.

The suggestion to include on the Committee of the Essex County Hospital two representatives of the Women's Committee has been carried. It arose out of an invitation to the working people of the town to support the hospital more generously than they had done hitherto. The recommendation made by the Hospital Committee that the income limit for out-patients, approved by the Governors at their meeting on February 26, 1909, be withdrawn, was also carried.

As the result of the recent match with Coventry, the entire gate-money of which was given by the Leicester Football Club to the Funds of the Infirmary, £115 8s. 4d. has been handed to the Treasurer. This brings the total contributions from the club to the institution to about £970. The Hon. President (Mr. J. Collier), Hon. Secretary (Mr. T. H. Crumbie), and Hon. Treasurer (Mr. F. St. Clair Pain) are already Life Governors of the Infirmary, and the board has invited the committee to nominate another member for election to a life governorship.

The Christmas entertainment for the patients of the East London Hospital for Children, Shadwell, E., will be held at the hospital on Friday, December 30, from 3 to 6 p.m.

The Queen has accepted a copy of Mr. Christopher Stone's "Lusus," which is being sold for the benefit of the Prince Francis of Teck Memorial Fund for the endowment of the Middlesex Hospital.

The annual meeting of the Mothers' Aid Society will be held at Queen Charlotte's Hospital, Marylebone Road (near Edgware Road Metropolitan Station), Wednesday, December 7, at 5 p.m. H.R.H. Princess Christian will take the chair. Tea at 4 p.m., after which some of the wards will be open for inspection before the meeting. Visitors are requested to be in their seats by 4.50 p.m.

The proceeds of the annual toy fair to be held on December 17 and 22, when the poorest fathers and mothers in the parish will come to buy ½d., 1d., and 2d. packets of cast-off toys as Christmas surprises for their own children, will be devoted to the provision of a roof-garden for St. Mary's Hospital for Women and Children. The address to which all presents of toys should be sent is the Rev. T. Given-Wilson, St. Mary's Mission Hall, Plaistow, E. All parcels should be marked "Toy Fair."

The new Theatre Royal, Windsor, is now completed, and will be opened on December 13. Mr. Frank Verity, F.R.I.B.A., is the architect. By invitation of the management the opening performances will be given by the Windsor Strollers, who will play "Under the Red Robe," "The Marriage of Kitty," and "The Sentence." Among the performers will be the Hon. S. Powys, Mr. Alan Mackinnon, Sir Spencer Ponsonby Fane, Lieut-Col. Newnham-Davis, and Miss Eve Benyon. All profits of these performances will be given to the King Edward VII. Hospital for Windsor, Eton, and District.

The yearly presentation of linen and other articles made to the Royal Victoria Hospital, Bournemouth, by the Ladies' Work Association, was made by the Countess of Malmesbury. The result of a year's work consisted of 214 sheets and draw sheets, 263 bolster and pillow cases, 109 damask tray and table cloths, 856 house cloths, 616 garments (flannel jackets, night clothes, children's clothes, etc.), and 300 towels, making a total of 2,358 articles. The total value of the linen, etc., made during the year, including blankets, quilts, waterproof sheeting, etc., bought with the subscriptions and donations was about £197 15s. Everyone felt keen regret at the absence through illness of the president of the association, Miss Wilkin.

### TO HOSPITAL SECRETARIES AND OTHERS.

We invite contributions from any of our readers, and shall be glad to pay, according to length, for items of news for the institutional section (including appointments, resignations, gifts, etc.) which we may publish. All payments for manuscripts used are made as early as possible after the beginning of each quarter.

Crowds of slum children are anxiously awaiting news from the Poor Children's Yule-Tide Association as to whether they are this year to be the happy recipients of a Christmas gift. Last year over 46,800 toys and 134 Christmas-trees were collected and distributed. It is earnestly hoped that some thousands more poor little slum children left out in 1909 will not be disappointed this season. It is now only a short time to Christmas, and I would most earnestly ask every reader of this appeal to post at once a subscription to the Hon. Secretary, 32 John Street, Theobald's Road, London, W.C.

### Gifts and Bequests.

The King has become an annual subscriber of £30 to the St. Andrew's Convalescent Hospital, Clewer.

Mrs. Mary Elizabeth Owen, of Llandovery, has bequeathed £500 to the Llandovery District Nursing Fund.

Leicester Infirmary has received £143 odd from the employés of the Enderby and Stoney Stanton Granite Co.

Mrs. Elizabeth Matthews, of Sunderland, Durham, has left £100 to the New Hospital for Children, Sunderland.

Miss Maria Furlong, of Highbury Crescent, Islington, left £1,000 to the Great Northern Central Hospital, London.

The Manchester Children's Hospital, Pendlebury, has received £50 from the Trustees of the late Mr. Frank Hardcastle, of Firwood, Bolton.

The King has made grants of £50 each to the St. Andrew's Convalescent Hospital, Clewer, and the Slough Nursing Home, from the Windsor Castle State Apartments Fund.

Miss Letitia Burrage, of Inverness Terrace, Bayswater, who died on September 5, aged ninety, has bequeathed £600 to the Medical Mission, Bell Street, Edgware Road.

Messrs. Joseph Stubbs and Co., Ltd., has forwarded £250 to Ancoats Hospital, Manchester, and the hon. treasurer has received £180 anonymously towards the appeal fund.

Mr. Frederick Satterthwaite, of Newlyn, Kilners Park, Ulverston, Lancashire, has bequeathed his estate (with the exception of certain furniture) with ultimate remainder to the Queen's Hospital for Children, Hackney Road, Bethnal Green.

Prince Alexander of Teck has received the following additional contributions to the Prince Francis of Teck Memorial Fund: By vote of the City Corporation, £105; Mr. W. C. Houghton, Mrs. Houghton, and Mr. Spencer Chapman, £50 each; Mr. Ernest Cunard, £20; the Naval and Military Club, £10; Canon Teignmouth Shore, £10; Mr. W. H. Kendal, £25; Mrs. Hope Vere, £20; Messrs. H. and C. Vivian (collection), £12; Captain Sir Anthony Abdy and Mrs. Seapher Stonhaugh, £10 each; Lord and Lady Ripon, £50; Mr. C. Birch Crisp, £52 10s.; Mrs. James H. Solomon, £50; "Anonymous," £25; and Sir W. E. Garstin and Mrs. F. Hemming, £10 each.

Lady Juliet Duff has received the following, among other contributions to the fund she is raising to free Charing Cross Hospital from debt: Mr. L. Neuman, £52 10s.; Mrs. Assheton Smith, £25; Hon. Walter and Lady Evelyn Guinness, £21; Mr. R. Manning Driver, £21; Mr. W. G. Vivian and Captain Herbert Wilson, £20 each; Mr. Perceval Landon and Mr. W. Koch, £10 10s. each; Mr. Charles Davis and Lord Crewe, £10 each.

Miss Emma Ann Corfield has bequeathed £500 to the North-Eastern Hospital for Children, Hackney Road, £400 each to the New Hospital for Women, Euston Road, and the Hospital for Sick Children, Great Ormond Street (for the convalescent branch), £400 to the Chelsea Hospital for Women, Fulham Road, £500 each to the London Hospital, Whitechapel, and King's College Hospital, and £500 to the National Hospital for Paralysed and Epileptics, Queen Square, Bloomsbury.

Miss Ivy Pick has sent a sum of £29 to the Leicester Infirmary as the result of dramatic performances recently. She has in previous years rendered valuable help, in recognition of which the Board has recommended her name for a Life Governorship.

Miss Catherine Munro Sutherland, of Newcastle-on-Tyne, has left £100 each to the Brunswick Wesleyan Nurses and Deaconesses' Society and the Royal Victoria Infirmary for Sick and Aged Poor of the counties of Northumberland and Durham.

Prince Arthur of Connaught has received the following in response to the appeal on behalf of the fund for new chemical laboratories at University College: Mr. Charles Hawksley, £250; Mr. Lewis Haslam, £100; Colonel Longstaff, £100; Messrs. H. K. Lewis, £100; Mr. Walter Morrison, £100; Sir Edward Tennant, £52 10s.; the Right Hon. Charles Booth, £50; Messrs. A. and F. Pears, Ltd., £26 5s.; Mr. Edward Filliter, £25; and Mr. Sigismund Goetze, £25.

### THE MEDICAL SCHOOLS.

The following are the arrangements for the week at the Central London Throat and Ear Hospital, Gray's Inn Road, W.C.: Lectures—Tuesday, December 13, at 3.45 p.m., "Accessory Services," Mr. W. Stuart-Low; Friday, December 16, at 3.45 p.m., "Larynx," Dr. Andrew Wylie.

The following are the arrangements for next week at the West London Post-Graduate College; December 12 and 15, at 10 a.m., demonstration by Surgical Registrar; December 16, at 10 a.m., demonstration by Medical Registrar; December 13, at 11.30 a.m., demonstration of minor operations; December 12, at 12 noon, pathological demonstration; December 14, at 12.15 p.m., lecture, Dr. Grainger Stewart, "Practical Medicine." Lectures at 5 p.m.: December 12, Mr. Armour, "Intestinal Obstruction"; December 13, Mr. Armour, "Intestinal Obstruction"; December 14, Mr. Lloyd Williams, "The Etiology, Prophylactics, and Treatment of Dental Caries"; December 15, Mr. Baldwin, "Practical Surgery"; December 16, Medical Registrar, "Examination of Abdomen."

The following are the arrangements at the Medical Graduates' College and Polyclinic, 22 Chenies Street, W.C.: Monday, December 12, at 4 p.m., Dr. J. M. H. MacLeod, Clinique (skin); at 5.15 p.m., Dr. William Ewart, "The Therapeutics of Bleeding." Tuesday, December 13, at 4 p.m., Dr. G. A. Sutherland, Clinique (medical); at 5.15 p.m., Dr. James Collier, "Some Forms of Peripheral Neuritis." Wednesday, December 14, at 4 p.m., Mr. G. E. Waugh, Clinique (surgical); at 5.15 p.m., Dr. W. S. A. Griffith, "The Use of the Douche." Thursday, December 15, at 4 p.m., Mr. H. J. Paterson, Clinique (surgical); at 5.15 p.m., Dr. J. Blumfeld, "Anæsthetics in Cases of Cardiac and Pulmonary Diseases." Friday, December 16, at 4 p.m., Dr. St. Clair Thomson, Clinique (ear, nose, and throat).

THE HOSPITAL December 10, 1910.

*Name* ..................................

*Address* ..................................

..................................

This Coupon must accompany manuscript or contributions intended for The Hospital.

Just Issued. Ninth Edition, Rewritten and Enlarged, 9s. net.

**PHARMACY, MATERIA MEDICA & THERAPEUTICS.**

Being a Commentary on the B.P. and its Drugs, with their Preparations, and on all the new Non-official Remedies in use, with Chapters on the different processes used in Dispensing, Prescription-writing, Latin Glossary, &c. By Sir W. WHITLA, M.D., LL.D., Professor, Materia Medica, Queen's University, Belfast, and Senior Physician, Royal Victoria Hospital.

By the same Author. New Work. 1,900 pages. Crown 8vo. 25s. net.

**PRACTICE OF MEDICINE.**

Containing in two Handy Volumes a complete and independent Article on the Etiology, Symptoms, and Diagnosis of every Medical Disease; arranged in Dictionary Form for Practitioners and Students.

London: Baillière, Tindall & Cox, 8 Henrietta Street, W.C.

A MANUAL OF

**MEDICAL EXERCISES.**

By PERCY LEWIS, M.D., M.R.C.S.,
Hon. Medical Officer to Victoria Hospital, Folkestone.

66 pages, with 35 Illustrations, cloth, 1s. 6d. net; post free, 1s. 7d.

H. K. LEWIS, 136 GOWER STREET, LONDON, W.C.

**Suggestions Invited.**

The Editor will welcome suggestions for the establishment of any new section in The Hospital, and will be glad to supply information on any subject of interest or importance to members of the profession in any part of the world.

**Correspondence.**

Correspondence on all subjects is invited, but no communication can be entertained if the name and address of the correspondent are not given as a guarantee of good faith, but not necessarily for publication All correspondents should write on one side [illegible] paper only.

### GENERAL PRACTITIONERS' CONTRIBUTIONS.

The Hospital devotes a special page to General Practitioners' contributions. We therefore invite from practitioners contributions based upon their experience in the management of cases, and in the treatment and diagnosis of disease; especially shall we be prepared to welcome articles dealing, practically, with treatment, and with the use and value of new remedies and methods.

No article should exceed 1,100 words, and, if accepted, one guinea for a contribution of this length will be paid to the writer after publication. Each communication should be accompanied by a stamped directed envelope for the return of the MS. if found unsuitable.

Contributions should be written, or preferably typed, on one side of the paper only, and all articles sent in are accepted upon the distinct understanding that they are forwarded to The Hospital only.

**The Relaxations of Medical Men.**

We shall also be glad to pay for accepted contributions, from any member of the profession, on the subject of the relaxations of practitioners. This opens up a wide field, as it includes natural history, photography, sport, indoor recreations, and motoring. Whenever possible, original illustrations and photographs should be sent with the MS.

**Books for Review.**

Publishers are particularly requested to send advance proofs of any new books of importance, whenever possible, as the Editor has made arrangements to publish immediate reviews, and on a new plan.

### BUSINESS NOTICES.

**Annual Subscriptions.**

The rate of annual subscriptions to The Hospital, either direct from the Office or through agents, is:—

| | | |
|---|---|---|
| For the United Kingdom ... ... | 15s. | a year. |
| For the Colonies and Abroad ... | 17s. | ,, |
| For the United Kingdom (with Insurance Policy) ... ... ... | £1 | ,, |

inclusive of postage in each case.

Letters relating to the Publishing, Sale and Advertisement Departments must be addressed to the Manager (*not to the Editor*):—

The Manager,
The Hospital Building,
28 and 29 Southampton Street,
Strand, London, W.C.

THE IDEAL WORK OF REFERENCE.

BURDETT'S HOSPITALS & CHARITIES.

*1910 EDITION.*

The Year-Book of Philanthropy and Hospital Annual.

Edited by SIR HENRY BURDETT, K.C.B., K.C.V.O.

Complete Guide and Directory of the Hospitals, Asylums, and Charitable Institutions throughout the English-speaking World.

Contains Special New Chapters on:—Voluntary Hospitals in 1909.—Soundness of the Voluntary System.—The need of greater zeal on the part of those who accept office.—State-aided Hospitals in the U.S.A. and Canada.—The Appropriation of Public Funds for the partial Support of Voluntary Hospitals in U.S.A. and Canada.—The Need of an Apostle and of greater interest on the part of the Clergy, &c., &c.

*TWENTY-FIRST YEAR OF PUBLICATION.*

Over 1,000 pages. Price 10/6 net; Post Free, 10/11.

ORDER your copy of the Medical Whitaker NOW.

Publishers: THE SCIENTIFIC PRESS, Ltd, 28 & 29 Southampton Street, Strand, London, W.C.

